# Investigation of Nursing Students' Online Information Searching Strategies and Attitudes Towards Informatics Ethical Values

**DOI:** 10.1111/scs.70020

**Published:** 2025-03-16

**Authors:** Necibe Dagcan Sahin, Gulsah Gurol Arslan, Dilek Ozden

**Affiliations:** ^1^ Faculty of Health Sciences Kutahya Health Sciences University Kutahya Turkey; ^2^ Faculty of Nursing Dokuz Eylul University Izmir Turkey

**Keywords:** informatics ethics, nursing student, online information search

## Abstract

**Aims and Objectives:**

Information technologies used in accessing online information also bring ethical problems. Informatics ethics are affected by students' socio‐demographic characteristics. This study was conducted to examine nursing students' online information searching strategies and attitudes towards informatics ethical values.

**Methods:**

A descriptive, cross‐sectional study design was employed. A non‐probability sampling method was used to determine the research sample. Data for the study were collected from students aged ≥ 18 years who were studying at the faculty of nursing of a university located in the west of the country between 15 September 2021 and 15 June 2022 and who agreed to participate in the research voluntarily. Data were collected using a ‘Descriptive Information Form’, the ‘Online Information Searching Strategy Inventory’ and the ‘Attitude Scale towards Informatics Ethical Values’.

**Results:**

Data were collected from a total of 710 first‐, second‐, third‐ and fourth‐year nursing students. Of the nursing students participating in the study, 40.1% were between the ages of 20 and 21, and 61.5% were female. A statistically significant difference was determined between their mean scores on the total information searching strategy scale and gender, class, the status of having a personal computer, level of computer use, the status of having an internet connection and the frequency of searching for information on the Internet (*p* < 0.05). In addition, there was a statistically significant difference between their mean scores on the total attitude scale towards informatics ethical values scale and age, gender, school year, the status of having a personal computer, the status of having an internet connection, frequency of searching for information on the Internet and the duration of searching for information online a week (*p* < 0.05). A positive, low‐level, statistically significant relationship was found between nursing students' attitudes towards informatics ethical values and online information searching strategies (*r* = 0.339, *p* = 0.000).

**Conclusion:**

In conclusion, it was found that when online information searching strategies increased, attitudes towards informatics ethical values increased as well. It is thought that our study results will be a guide for nursing students to develop online information search strategies throughout their educational lives and raise awareness about informatics ethics. It may be recommended that students' information search strategies be determined, information sources be found, information be used and necessary arrangements be made to meet their needs.

## 1 Introduction

Today, many people use the Internet to get information thanks to developing science and technology, and it is one of the easiest ways to access information [1, 2]. Developments in technology also affect health information systems and the delivery of healthcare [3]. Information and communication technologies are used to improve health and prevent and treat diseases [4]. They are also utilised in practical clinical training and theoretical education in nursing, which is an important field of health [3, 4, 5].

## 2 Background

The discipline of nursing focuses on human health, healing and care, and is comprised of elements, such as science, art, philosophy and ethics [6]. Models and theories provide the scientific knowledge base that guides nursing practice and contributes to the development of the discipline. Knowing how the concepts of nursing and care are related by theorists or modellers provides an understanding of the evolution of care over time [7]. Watson stated in the late 1970s and 1980s that caring, an indicator of professional nursing, was the fifth core concept of nursing [7]. According to Watson (1989), care is a professional interaction process based on trust and respect between two people, with its ethical, scientific and aesthetic aspects [8]. Nurses should have ethical responsibility in the care process. According to Paul Ricoeur, ethics is related to the individual living a good life, which requires nurses to consider the well‐being of patients in care [9, 10].

For nursing students, the ability to relate ethics to theories of care helps them develop a professional perspective. Nursing education should guide students in the ethical use of digital health applications and teach them how to use these technologies ethically in patient care. Students should make the right decisions in accordance with the principles of informatics ethics and use technology in a way that will improve the quality of patient care [11, 12]. In this context, the relationship between informatics ethics and nursing care theories is thought to be important in how nurses and students fulfil their ethical responsibilities when using digital tools.

Nursing informatics, which is the use of information technologies in nursing‐specific knowledge and application areas, began to develop rapidly about 10 years ago [2, 3]. It provides support in the evaluation and development of patient care and the tools used in care through data management [2, 5]. With the development of information technologies in nursing, the accumulation and transfer of knowledge have augmented [4]. Every nurse must have knowledge about information technologies and integrate the relevant competencies into their profession [2, 3].

Developing information technologies allow rapid access to information, and they are often used in the field of 21st‐century education in this respect [4, 13]. The Internet facilitates learning as it provides the flexibility of time and place [1]. Students search for information online for their studies and assignments [2, 13]. Their online information searching strategies are very important in obtaining accurate information [2]. Online information search strategies include quickly accessing accurate and reliable information on the Internet, analysing this information, evaluating it and making decisions [14]. Having the ability to search for information using the Internet allows for accessing, evaluating and utilising information effectively [13]. When the literature is examined, the study results reporting that online information search strategies are affected by socio‐demographic characteristics stand out [15]. For nursing students, who will be the nurses of tomorrow, it is of great importance that they access online information easily and quickly, decide on the accuracy of the information they obtain and evaluate and analyse it [14]. It is thought that students can increase their motivation by ensuring that the care they plan for a healthy patient individual reaches their goals. It is reported in the literature that factors, such as knowledge and experience, competence and participation in decisions affect motivation, quality of care and professional development of nurses [16]. This shows the importance of students' online information searching strategies.

Information technologies used in accessing online information also bring ethical problems. Research into these problems dates back to the year 2000 [17, 18]. Considering the diversity of information obtained, its reliability, accuracy and objectivity become a major issues [19]. Informatics ethics is a sub‐dimension of ethics that includes the rules that must be followed when using information technology tools to prevent users from harm [20]. With the use of digital technologies in healthcare, informatics has begun to play an important role in nursing practice [4, 21]. Although there are principles and rules for informatics ethics today, there are also gaps and individuals may face problems, such as the privacy of their lives, accuracy of information, fraud, virtual relationships and security [22]. Although digital tools, such as electronic health records and mobile health applications, bring innovation to the care process, they also raise ethical issues, such as the protection of patient data, the ethical use of technology and the new responsibilities that nurses face in the digital environment [4, 21, 23, 24]. Students who spend too much time on technology may have difficulty accessing accurate information despite their awareness [25]. For this reason, it is thought that nursing students should be ready for the world of digital health. Informatics ethics has an important place in preparing students for the difficulties they may encounter in real life and is also affected by students' socio‐demographic characteristics [13, 17]. Yanar et al. [26] report that there is a relationship between attitudes towards ethical values in informatics and age. Kurt and Teker [27] found that ethical behaviour scores increase as students' school years increase. Again, there are study results that mention the relationship between gender and informatics ethics [27]. Accordingly, it is of great importance to determine students' ethical perceptions towards problems in the online environment.

No studies have been found in the literature on online information search and information ethics. It is thought that student nurses' awareness of the ethical use of digital health technologies will increase the quality of care and contribute to their professional development by providing safe patient care. In this respect, our study findings can fill the gap in the literature and contribute.

## 3 Aim

A review of the literature indicated that there was no study on the examination of nursing students' online information searching strategies and attitudes towards informatics ethical values. Our study aimed to reveal nursing students' online information searching strategies and attitudes towards informatics ethical values and to explain the relationship between the two.

## 4 Design

### 4.1 Methods

This study was planned in a cross‐sectional‐descriptive design. The equator checklist document used in this study was the Strengthening the Reporting of Observational Studies in Epidemiology (STROBE). In order to increase the sample size as much as possible in the study, the non‐probability convenience sampling method was chosen. The aim of this method was to include everyone who wants to be included in the sample. The process of finding subjects continues until the determined sample size is reached. This method provides convenience in terms of both time and economy. However, it also limits the generalisability of the study [28]. For this reason, in our study, we tried to reach all first, second, third and fourth grade students who were 18 years of age and older, students in the nursing department and volunteered to participate in the study.

### 4.2 Participants and Research Context

The population of the research consisted of a total of 1039 students, including 252 freshmen, 256 sophomores, 278 juniors and 253 seniors, studying at the nursing faculty of a university located in the west of the country in the 2021–2022 academic year. The data of the study were collected from 710 students who were aged ≥ 18 years and voluntarily agreed to participate in this study between 15 September 2021 and 15 June 2022.

### 4.3 Outcome Measures

#### 4.3.1 Descriptive Characteristics Form

This form was created by the researcher following a review of the literature [1, 2, 14] and consisted of a total of 12 questions about students' demographic and individual characteristics, such as age, gender and school year.

#### 4.3.2 Online Information Searching Strategy Inventory (OISSI)

This scale was developed by Tsai [29]. Its adaptation to Turkish and the validity and reliability study of the Turkish form were conducted by Askar and Mazman Akar [20]. The scale consists of 25 items and a 6‐point Likert‐type structure to evaluate the items. Each item on the scale is evaluated with a score between 1 and 6. The scale is a 6‐point Likert‐type scale ranging from ‘It does not apply to me at all’ to ‘It completely applies to me’. The scale is used to determine individuals' information search strategies in online environments and the responses range from ‘It does not apply to me at all’ to ‘It completely applies to me’. The scale includes seven sub‐dimensions: ‘Disorientation’, ‘Evaluation’, ‘Purposeful Thinking’, ‘Trial and Error’, ‘Basic ideas differentiation’, ‘Control’ and ‘Problem Solving’. Scores on the scale range between 25 and 150. A high total score indicates having a high level of online information searching strategy. Cronbach's alpha coefficient of the scale was determined as 0.910 [20]. The alpha value was found as 0.924 in this study.

#### 4.3.3 Attitude Scale Towards Informatics Ethical Values (ASIEV)

This scale was created by Celik and Gundogdu [18]. It has 31 items and a four‐point Likert‐type structure with options ranging from ‘strongly disagree’ to ‘strongly agree’. Reverse coded items are 7–22. The scale has eight dimensions, including Virtual helpfulness, Violation of privacy, Respect for copyright, Virtual bullying, Security, Virtual media collaboration, Virtual ethics and Virtual sharing. Reliability coefficients in the scale were found to be 0.875 for virtual benevolence, 0.925 for privacy violation, 0.820 for copyright respect, 0.692 for cyberbullying, 0.570 for security, 0.801 for cybercollaboration, 0.860 for cybermorality and 0.650 for social sharing. Cronbach's alpha reliability coefficient for the total scale was found to be 0.798 [18]. The alpha value was calculated as 0.807 in this study.

### 4.4 Data Collection

Research data were collected from first‐, second‐, third‐ and fourth‐year students studying at the nursing faculty of a university located in the 2021–2022 academic year. Before the research was initiated, students who met the inclusion criteria were informed about the purpose of the study and the scales to be used, and their verbal and written consent was obtained. Then, they filled out the Descriptive Characteristics Form, the OISSI and the ASIEV. The researcher accompanied the students during the data collection process.

### 4.5 Data Analysis

The study data were analysed by the researcher using the SPSS 26.0 software. Frequency, percentage, arithmetic mean values, and Cronbach's alpha reliability coefficient were calculated to analyse the data. Percentages were used to evaluate students' descriptive characteristics. The normality assumption was tested with the Shapiro–Wilk test. It was determined that the data conformed to a normal distribution (*p* < 0.05). According to this analysis, the differences between the independent variables and the total scale scores with a normal distribution were examined using the student's *t*‐test and one‐way analysis of variance (ANOVA), which are parametric tests. Results were given as mean ± standard deviation values. *p* < 0.05 was considered statistically significant. Means, medians and lower‐upper values were used for descriptive variables specified by measurement, and numbers and percentages were used for variables specified by count.

### 4.6 Ethical Considerations

Approval was received from the faculty where the research was conducted and from the ethics committee. In addition, participants who met the inclusion criteria were informed about the study, and their consent was obtained. Verbal and written consent was obtained from the participants. The participants were informed that the data obtained would be used for scientific purposes and that no information regarding the data would be presented anywhere. In addition, the surveys obtained are protected by the first researcher. Participants' personal information was kept anonymous and not disclosed to anyone. In addition, the data were kept private and confidential. All procedures were in accordance with the ethical standards of the institutional and/or national research committee and with the 1964 Helsinki Declaration and its later amendments or comparable ethical standards.

## 5 Results

The evaluation of students' demographic characteristics showed that 40.1% were aged between 20 and 21, 61.5% were female, 29% were first‐year students, 80.6% were Anatolian high school graduates, 49.2% had equal income and expenses, 51.7% had a personal computer, 45.8% had a moderate level of computer use, 85.9% had an internet connection, the duration of daily internet use was 2–3 h in 37.6% and 4–5 h in 37.5%, 69.7% searched for information on the Internet every day, and 62.1% spent 0–7 h searching for information online a week (Table 1).

**TABLE 1:** Nursing students' demographic characteristics (*n* = 710).

Variables	*n*	%
Age (years)		
18–19	188	26.5
20–21	285	40.1
≥ 22	237	33.4
Mean ± SD	20.86 ± 2.31	
Gender		
Female	437	61.5
Male	273	38.5
School year		
1	206	29.0
2	185	26.1
3	151	21.3
4	168	23.7
High school		
Anatolian high school	572	80.6
General high school	45	6.3
Technical and vocational high school	40	5.6
Others	53	7.5
Income		
Income > expenses	304	42.8
Income = expenses	349	49.2
Income < expenses	57	8.0
Status of having a personal computer		
Yes	367	51.7
No	343	48.3
Level of computer use		
None	23	3.2
Low	153	21.5
Moderate	325	45.8
Good	156	22.0
Very good	53	7.5
Status of having an internet connection		
Yes	610	85.9
No	100	14.1
Duration of daily internet use (hours)		
0–1	37	5.2
2–3	267	37.6
4–5	266	37.5
≥ 6	140	19.7
Frequency of searching for online information		
Every day	495	69.7
Several days a week	177	24.9
Once a week	26	3.7
Once a month	12	1.7
Duration of weekly online information searching (hour)		
0–7	441	62.1
8–14	182	25.6
≥ 15	87	12.3

The distribution of nursing students' mean scores on the total and sub‐dimensions of the online information searching strategy scale according to their demographic characteristics indicated that there was a statistically significant difference in the scores on the total (*p* = 0.013) and the disorientation (*p* = 0.005), trial and error (*p* = 0.000), and problem‐solving (*p* = 0.034) sub‐dimensions of the online information searching strategy scale according to gender. A statistically significant difference was found in the students' total mean scores on the online information search strategy scale according to their personal computer ownership (*p* = 0.002), computer usage level (*p* = 0.000), internet connection (*p* = 0.000) and frequency of searching for information on the internet (*p* = 0.000) (Table 2).

**TABLE 2:** Distribution of nursing students' mean scores on the online information searching strategy scale and its sub‐dimensions according to their demographic characteristics (*n* = 710).

Variables	OISSI							
	Disorientation	Evaluation	Purposeful thinking	Trial and error	Basic ideas differentiation	Control	Problem‐solving	Total
	Mean ± SD	Mean ± SD	Mean ± SD	Mean ± SD	Mean ± SD	Mean ± SD	Mean ± SD	Mean ± SD
Age (years)								
18–19	9.73 ± 4.05	17.04 ± 3.91	17.48 ± 4.05	13.61 ± 3.59	13.57 ± 3.21	17.27 ± 3.76	13.31 ± 2.92	110.55 ± 19.88
20–21	9.71 ± 3.54	17.23 ± 4.19	17.44 ± 4.16	13.60 ± 3.38	13.42 ± 3.36	17.04 ± 4.27	13.10 ± 2.71	110.12 ± 20.03
≥ 22	10.14 ± 4.16	16.99 ± 4.15	17.31 ± 3.97	13.08 ± 3.45	13.13 ± 3.37	17.02 ± 4.24	12.95 ± 2.80	108.33 ± 20.25
Test	*f* = 0.921 *p* = 0.399	*f* = 0.254 *p* = 0.776	*f* = 0.106 *p* = 0.900	*f* = 1.796 *p* = 0.167	*f* = 1.022 *p* = 0.361	*f* = 0.222 *p* = 0.801	*f* = 0.862 *p* = 0.423	*f* = 0.777 *p* = 0.460
Gender								
Female	9.54 ± 3.899	17.27 ± 4.076	17.56 ± 4.044	13.81 ± 3.386	13.47 ± 3.297	17.27 ± 4.067	13.28 ± 2.770	111.12 ± 19.688
Male	10.38 ± 3.837	16.82 ± 4.140	17.16 ± 4.102	12.82 ± 3.518	13.19 ± 3.374	16.82 ± 4.227	12.82 ± 2.829	107.27 ± 20.462
Test	***t* = −2.816** ***p* = 0.005**	*t* = 1.417 *p* = 0.157	*t* = 1.259 *p* = 0.209	***t* = 3.701** ***p* = 0.000**	*t* = 1.095 *p* = 0.274	*t* = 1.397 *p* = 0.163	***t* = 2.122** ***p* = 0.034**	***t* = 2.496** ***p* = 0.013**
School year								
1	9.49 ± 3.787	16.92 ± 4.133	17.11 ± 4.268	13.66 ± 3.590	13.46 ± 3.309	17.06 ± 3.949	13.11 ± 2.946	109.82 ± 19.626
2	9.81 ± 3.887	17.82 ± 3.735	18.23 ± 3.693	14.11 ± 3.168	14.18 ± 3.039	17.57 ± 4.240	13.68 ± 2.498	113.78 ± 19.337
3	10.68 ± 4.109	16.51 ± 4.178	17.12 ± 4.131	12.62 ± 3.390	12.70 ± 3.337	16.58 ± 4.006	12.46 ± 2.715	105.30 ± 19.172
4	9.63 ± 3.760	17.06 ± 4.305	17.11 ± 4.065	13.13 ± 3.546	12.95 ± 3.472	17.08 ± 4.317	13.05 ± 2.897	108.74 ± 21.377
Test	*f* = 3.056 *p* = 0.028	*f* = 3.117 *p* = 0.026	*f* = 3.493 *p* = 0.015	*f* = 6.018 *p* = 0.000	*f* = 6.800 *p* = 0.000	*f* = 1.609 *p* = 0.186	*f* = 5.372 *p* = 0.001	*f* = 5.190 *p* = 0.001
High school								
Anatolian	9.80 ± 3.861	17.06 ± 4.116	17.46 ± 4.078	13.47 ± 3.455	13.37 ± 3.267	17.09 ± 4.110	13.10 ± 2.754	109.75 ± 19.891
General	10.87 ± 4.490	16.27 ± 3.968	16.84 ± 4.073	12.84 ± 3.384	13.44 ± 3.507	16.42 ± 4.081	12.53 ± 2.817	105.49 ± 20.052
Technical and vocational	9.70 ± 4.084	18.43 ± 3.700	17.75 ± 3.593	13.85 ± 3.613	13.88 ± 3.480	17.85 ± 4.234	13.48 ± 2.755	113.53 ± 19.983
Others	9.75 ± 3.540	17.25 ± 4.238	17.04 ± 4.341	13.13 ± 3.584	12.83 ± 3.704	17.15 ± 4.347	13.32 ± 3.286	108.96 ± 21.828
Test	*f* = 1.082 *p* = 0.356	*f* = 2.059 *p* = 0.104	*f* = 0.556 *p* = 0.644	*f* = 0.781 *p* = 0.505	*f* = 0.778 *p* = 0.506	*f* = 0.846 *p* = 0.469	*f* = 0.963 *p* = 0.410	*f* = 1.169 *p* = 0.321
Income								
> expenses	10.25 ± 3.943	17.12 ± 4.283	17.52 ± 4.155	13.62 ± 3.442	13.46 ± 3.399	16.93 ± 4.338	13.13 ± 2.826	109.53 ± 20.858
= expenses	9.53 ± 3.786	17.02 ± 3.941	17.18 ± 4.005	13.23 ± 3.491	13.25 ± 3.302	17.17 ± 3.867	13.12 ± 2.767	109.44 ± 19.335
< expenses	9.79 ± 4.156	17.51 ± 4.150	18.12 ± 3.942	13.65 ± 3.451	13.53 ± 3.112	17.49 ± 4.591	12.91 ± 2.905	111.42 ± 20.376
Test	*f* = 2.800 *p* = 0.061	*f* = 0.356 *p* = 0.700	*f* = 1.535 *p* = 0.216	*f* = 1.132 *p* = 0.323	*f* = 0.411 *p* = 0.663	*f* = 0.548 *p* = 0.579	*f* = 0.149 *p* = 0.862	*f* = 0.247 *p* = 0.782
Status of having a personal computer								
Yes	9.18 ± 3.834	17.52 ± 4.018	17.58 ± 4.099	13.51 ± 3.527	13.40 ± 3.335	17.72 ± 4.085	13.29 ± 2.872	111.84 ± 19.948
No	10.59 ± 3.831	16.65 ± 4.151	17.22 ± 4.033	13.34 ± 3.406	13.33 ± 3.323	16.42 ± 4.082	12.91 ± 2.712	107.28 ± 19.945
Test	***t* = −4.905** ***p* = 0.000**	***t* = 2.838** ***p* = 0.005**	*t* = 1.194 *p* = 0.233	*t* = 0.646 *p* = 0.518	*t* = 0.285 *p* = 0.776	***t* = 4.237** ***p* = 0.000**	*t* = 1.779 *p* = 0.076	***t* = 3.044** ***p* = 0.002**
Level of computer use								
None	10.61 ± 4.500	15.39 ± 5.408	16.30 ± 4.685	3.843 ± 0.801	12.04 ± 4.497	14.30 ± 4.587	12.83 ± 2.462	100.22 ± 23.440
Low	10.47 ± 3.744	16.01 ± 4.160	16.61 ± 4.258	3.566 ± 0.288	12.97 ± 3.335	15.59 ± 4.035	12.39 ± 2.916	103.83 ± 20.317
Moderate	10.22 ± 3.815	16.78 ± 3.675	17.15 ± 3.706	3.200 ± 0.178	13.19 ± 3.102	16.87 ± 3.746	12.95 ± 2.646	107.98 ± 17.970
Good	8.88 ± 3.821	18.17 ± 4.017	18.10 ± 4.056	3.489 ± 0.279	13.86 ± 3.293	18.43 ± 4.007	13.71 ± 2.712	115.66 ± 19.663
Very good	8.42 ± 3.983	19.83 ± 4.232	19.68 ± 4.389	3.795 ± 0.521	14.70 ± 3.688	20.08 ± 4.028	14.51 ± 3.023	122.94 ± 20.958
Test	***f* = 6.355** ***p* = 0.000**	***f* = 13.592** ***p* = 0.000**	***f* = 7.763** ***p* = 0.000**	***f* = 6.741** ***p* = 0.000**	***f* = 4.771** ***p* = 0.000**	***f* = 20.995** ***p* = 0.000**	***f* = 8.290** ***p* = 0.000**	***f* = 15.553** ***p* = 0.000**
Status of having an internet connection								
Yes	9.68 ± 3.873	17.29 ± 4.091	17.61 ± 4.002	13.57 ± 3.436	13.48 ± 3.262	17.34 ± 4.106	13.26 ± 2.809	110.86 ± 19.737
No	10.94 ± 3.866	15.96 ± 4.012	16.17 ± 4.269	12.57 ± 3.554	12.65 ± 3.639	15.58 ± 3.985	12.18 ± 2.572	102.17 ± 20.514
Test	***t* = −3.011** ***p* = 0.003**	***t* = 3.014** ***p* = 0.003**	***t* = 3.296** ***p* = 0.001**	***t* = 2.682** ***p* = 0.007**	***t* = 2.320** ***p* = 0.021**	***t* = 3.996** ***p* = 0.000**	***t* = 3.596** ***p* = 0.000**	***t* = 4.059** ***p* = 0.000**
Duration of daily internet use (hour)								
0–1	10.49 ± 3.962	16.89 ± 4.465	16.62 ± 4.493	12.81 ± 3.872	13.08 ± 3.531	16.57 ± 4.658	12.76 ± 3.312	106.24 ± 23.750
2–3	10.16 ± 3.835	16.93 ± 4.002	17.42 ± 4.055	13.32 ± 3.439	13.25 ± 3.347	16.81 ± 4.060	13.17 ± 2.773	108.73 ± 20.155
4–5	9.53 ± 3.842	17.02 ± 4.034	17.61 ± 3.809	13.56 ± 3.261	13.42 ± 3.208	17.22 ± 3.931	13.14 ± 2.682	110.44 ± 18.631
≥ 6	9.74 ± 4.061	17.64 ± 4.320	17.19 ± 4.446	13.55 ± 3.792	13.53 ± 3.477	17.55 ± 4.471	13.01 ± 2.944	110.73 ± 21.465
Test	*f* = 1.533 *p* = 0.205	*f* = 1.045 *p* = 0.372	*f* = 0.815 *p* = 0.486	*f* = 0.652 *p* = 0.582	*f* = 0.328 *p* = 0.805	*f* = 1.285 *p* = 0.279	*f* = 0.309 *p* = 0.819	*f* = 0.816 *p* = 0.485
Frequency of searching for online information								
Every day	9.68 ± 3.818	17.60 ± 4.045	17.72 ± 4.027	13.81 ± 3.360	13.74 ± 3.214	17.40 ± 4.078	13.36 ± 2.744	111.96 ± 19.706
Several days a week	10.03 ± 3.966	16.19 ± 3.999	16.94 ± 4.011	12.75 ± 3.643	12.77 ± 3.343	16.58 ± 4.150	12.86 ± 2.809	106.06 ± 19.730
Once a week	11.58 ± 4.941	15.35 ± 3.888	15.92 ± 4.137	12.15 ± 2.976	11.50 ± 3.744	16.15 ± 4.540	11.15 ± 2.708	98.65 ± 20.558
Once a month	10.75 ± 2.417	13.67 ± 3.892	14.25 ± 4.288	10.42 ± 2.811	10.50 ± 3.119	14.08 ± 3.260	10.42 ± 1.881	90.58 ± 13.608
Test	*f* = 2.359 *p* = 0.070	***f* = 10.157** ***p* = 0.000**	***f* = 5.424** ***p* = 0.001**	***f* = 8.774** ***p* = 0.000**	***f* = 10.032** ***p* = 0.000**	***f* = 4.481** ***p* = 0.004**	***f* = 10.067** ***p* = 0.000**	***f* = 10.711** ***p* = 0.000**
Duration of weekly online information searching (hour)								
0–7	9.93 ± 3.766	16.83 ± 4.028	17.24 ± 4.079	13.21 ± 3.530	13.18 ± 3.386	16.75 ± 4.162	12.97 ± 2.831	108.25 ± 20.166
8–14	9.71 ± 4.265	17.54 ± 4.237	17.77 ± 4.032	13.70 ± 3.437	13.55 ± 3.260	17.60 ± 4.173	13.45 ± 2.764	111.90 ± 20.450
≥ 15	9.78 ± 3.755	17.54 ± 4.125	17.46 ± 4.089	13.98 ± 3.133	13.90 ± 3.114	17.77 ± 3.728	13.08 ± 2.686	111.94 ± 18.206
Test	*f* = 0.225 *p* = 0.799	*f* = 2.532 *p* = 0.080	*f* = 1.089 *p* = 0.337	*f* = 2.576 *p* = 0.077	*f* = 2.068 *p* = 0.127	***f* = 4.062** ***p* = 0.018**	*f* = 1.858 *p* = 0.157	*f* = 2.797 *p* = 0.062

Abbreviations: f, ANOVA; *t*, *t*‐test. The significant values bold represent (p < 0.05).

As a result of examining the total mean scores of the attitude scale towards informatics ethical values of nursing students according to demographic characteristics, a statistically significant difference was found in the mean scores of the scale according to age (*p* = 0.001), gender (*p* = 0.000), school year (*p* = 0.000), personal computer ownership (*p* = 0.000), internet connection ownership (*p* = 0.030), frequency of searching for information on the internet (*p* = 0.011) and weekly online information searching time variable (*p* = 0.000) (Table 3).

**TABLE 3:** Distribution of nursing students' mean scores on the total and sub‐dimensions of the attitudes towards informatics ethical values scale according to their demographic characteristics (*n* = 710).

Variables	ASIEV								
	Virtual helpfulness	Violation of privacy	Respect for copyright	Virtual bullying	Security	Virtual media collaboration	Virtual ethics	Virtual sharing	Total
	Mean ± SD	Mean ± SD	Mean ± SD	Mean ± SD	Mean ± SD	Mean ± SD	Mean ± SD	Mean ± SD	Mean ± SD
Age (years)									
18–19	12.44 ± 3.942	6.63 ± 3.097	10.31 ± 2.315	4.26 ± 1.888	9.79 ± 1.857	10.29 ± 2.60	13.62 ± 2.82	8.31 ± 2.046	93.89 ± 10.0
20–21	12.88 ± 4.668	7.01 ± 3.438	10.44 ± 2.195	4.14 ± 1.958	9.86 ± 1.960	10.68 ± 2.71	13.11 ± 3.29	8.47 ± 2.349	94.27 ± 11.0
≥ 22	12.30 ± 4.409	7.55 ± 3.990	9.84 ± 2.680	4.44 ± 2.126	9.59 ± 2.293	10.68 ± 2.77	12.38 ± 3.38	8.09 ± 2.418	90.90 ± 11.2
Test	*f* = 1.241 *p* = 0.290	***f* = 3.680** ***p* = 0.026**	***f* = 4.233** ***p* = 0.015**	*f* = 1.457 *p* = 0.234	*f* = 1.098 *p* = 0.334	*f* = 1.417 *p* = 0.243	***f* = 8.133** ***p* = 0.000**	*f* = 1.718 *p* = 0.180	***f* = 7.020** ***p* = 0.001**
Gender									
Female	12.69 ± 4.250	6.64 ± 3.208	10.78 ± 2.110	3.99 ± 1.769	9.96 ± 1.911	10.56 ± 2.8	13.44 ± 3.16	8.27 ± 2.276	95.08 ± 10.3
Male	12.38 ± 4.633	7.81 ± 3.969	9.29 ± 2.573	4.73 ± 2.251	9.41 ± 2.223	10.61 ± 2.5	12.29 ± 3.22	8.35 ± 2.339	89.80 ± 11.1
Test	*t* = 0.920 *p* = 0.358	***t* = −4.305** ***p* = 0.000**	***t* = 8.408** ***p* = 0.000**	***t* = −4.862** ***p* = 0.000**	***t* = 3.498** ***p* = 0.000**	*t* = −0.266 *p* = 0.790	*t* **= 4.690** ***p* = 0.000**	*t* = −0.460 *p* = 0.646	***t* = 6.430** ***p* = 0.000**
School year (years)									
1	12.59 ± 4.226	7.19 ± 3.530	10.02 ± 2.363	4.52 ± 2.092	9.67 ± 1.985	10.25 ± 2.6	13.00 ± 3.11	8.07 ± 2.136	91.89 ± 10.8
2	12.92 ± 4.463	6.17 ± 2.524	10.81 ± 1.971	3.84 ± 1.512	9.91 ± 1.838	10.59 ± 2.5	13.91 ± 2.98	8.75 ± 2.200	96.88 ± 9.38
3	13.04 ± 4.572	8.28 ± 4.338	9.53 ± 2.797	4.77 ± 2.507	9.33 ± 2.371	10.83 ± 2.6	11.97 ± 3.15	8.26 ± 2.374	89.91 ± 11.7
4	11.75 ± 4.307	6.90 ± 3.522	10.38 ± 2.364	4.00 ± 1.688	10.06 ± 1.99	10.73 ± 2.8	12.92 ± 3.45	8.13 ± 2.474	93.07 ± 10.7
Test	***f* = 2.931** ***p* = 0.033**	***f* = 10.360** ***p* = 0.000**	***f* = 8.747** ***p* = 0.000**	***f* = 8.317** ***p* = 0.000**	***f* = 3.888** ***p* = 0.009**	*f* = 1.618 *p* = 0.184	***f* = 10.470** ***p* = 0.000**	***f* = 3.448** ***p* = 0.016**	***f* = 13.097** ***p* = 0.000**
High school									
Anatolian	12.38 ± 4.380	6.91 ± 3.405	10.33 ± 2.371	4.19 ± 1.957	9.82 ± 1.997	10.45 ± 2.7	13.20 ± 3.15	8.34 ± 2.295	93.43 ± 10.7
General	13.47 ± 4.197	7.89 ± 4.255	9.76 ± 2.604	4.82 ± 2.358	9.36 ± 2.308	10.89 ± 2.8	12.33 ± 3.78	8.31 ± 2.391	91.40 ± 11.9
Technical and vocational	13.60 ± 4.845	8.05 ± 4.242	9.18 ± 2.297	4.65 ± 1.819	9.30 ± 2.312	11.08 ± 2.6	11.73 ± 3.47	7.53 ± 2.407	89.70 ± 11.476
Others	13.13 ± 4.319	7.64 ± 3.873	10.00 ± 2.542	4.43 ± 2.188	9.70 ± 2.189	11.34 ± 2.1	12.32 ± 3.23	8.43 ± 2.135	92.85 ± 10.9
Test	*f* = 2.008 *p* = 0.112	***f* = 2.655** ***p* = 0.048**	***f* = 3.674** ***p* = 0.012**	*f* = 2.054 *p* = 0.105	*f* = 1.430 *p* = 0.233	*f* = 2.502 *p* = 0.058	***f* = 4.302** ***p* = 0.005**	*f* = 1.645 *p* = 0.178	*f* = 1.833 *p* = 0.140
Income									
> expenses	12.65 ± 4.499	7.32 ± 3.539	10.05 ± 2.444	4.55 ± 2.192	9.58 ± 2.119	10.52 ± 2.6	12.91 ± 13.1	8.30 ± 2.142	92.16 ± 11.1
= expenses	12.27 ± 4.190	6.67 ± 3.275	10.41 ± 2.312	4.02 ± 1.736	9.91 ± 1.940	10.65 ± 2.6	13.14 ± 3.32	8.31 ± 2.409	94.00 ± 10.3
< expenses	14.02 ± 4.883	8.44 ± 4.803	9.82 ± 2.720	4.37 ± 2.249	9.70 ± 2.307	10.40 ± 3.3	12.58 ± 3.00	8.23 ± 2.457	91.95 ± 12.9
Test	***f* = 4.004** ***p* = 0.019**	***f* = 7.204** ***p* = 0.001**	*f* = 2.582 *p* = 0.076	***f* = 5.842** ***p* = 0.003**	*f* = 2.154 *p* = 0.117	*f* = 0.315 *p* = 0.730	*f* = 0.930 *p* = 0.395	*f* = 0.033 *p* = 0.968	*f* = 2.630 *p* = 0.073
Status of having a personal computer									
Yes	12.60 ± 4.449	6.71 ± 3.369	10.29 ± 2.367	4.01 ± 1.699	9.96 ± 1.915	10.86 ± 2.6	13.04 ± 3.24	8.19 ± 2.338	94.23 ± 10.6
No	12.54 ± 4.354	7.50 ± 3.721	10.12 ± 2.454	4.56 ± 2.244	9.52 ± 2.169	10.27 ± 2.7	12.96 ± 3.23	8.42 ± 2.254	91.78 ± 11.0
Test	*t* = 0.198 *p* = 0.843	***t* = −2.948** ***p* = 0.003**	*t* = 0.967 *p* = 0.334	***t* = −3.707** ***p* = 0.000**	***t* = 2.868** ***p* = 0.004**	***t* = 2.901** ***p* = 0.004**	*t* = 0.325 *p* = 0.746	*t* = −1.295 *p* = 0.196	***t* = 3.011** ***p* = 0.003**
Level of computer use									
None	12.61 ± 5.106	7.96 ± 4.810	9.91 ± 2.745	4.57 ± 2.483	10.22 ± 1.75	9.22 ± 3.24	13.57 ± 2.77	8.48 ± 2.678	91.48 ± 8.85
Low	11.93 ± 4.469	6.90 ± 3.107	10.32 ± 2.364	4.31 ± 1.985	9.65 ± 1.941	10.32 ± 2.4	12.99 ± 3.36	8.53 ± 2.320	92.53 ± 11.0
Moderate	12.40 ± 4.191	6.93 ± 3.356	10.38 ± 2.355	4.21 ± 1.882	9.82 ± 1.970	10.60 ± 2.7	13.17 ± 3.18	8.22 ± 2.209	93.46 ± 10.5
Good	13.23 ± 4.201	7.10 ± 3.745	10.10 ± 2.367	4.23 ± 2.063	9.76 ± 2.086	11.13 ± 2.5	12.75 ± 3.16	8.40 ± 2.325	94.05 ± 11.0
Very good	13.51 ± 5.384	8.23 ± 4.602	9.26 ± 2.676	4.57 ± 2.333	9.36 ± 2.767	10.15 ± 2.9	12.47 ± 3.57	7.79 ± 2.507	89.75 ± 12.8
Test	*f* = 2.417 ***p* = 0.047**	*f* = 1.978 *p* = 0.096	*f* = 2.711 ***p* = 0.029**	*f* = 0.522 *p* = 0.720	*f* = 0.973 *p* = 0.421	*f* = 3.838 ***p* = 0.004**	*f* = 0.991 *p* = 0.412	*f* = 1.253 *p* = 0.287	*f* = 1.856 *p* = 0.116
Status of having an internet connection									
Yes	12.55 ± 4.432	6.96 ± 3.485	10.24 ± 2.412	4.20 ± 1.954	9.76 ± 2.042	10.60 ± 2.7	13.07 ± 3.21	8.35 ± 2.288	93.41 ± 10.9
No	12.71 ± 4.219	7.86 ± 3.936	10.02 ± 2.395	4.75 ± 2.204	9.69 ± 2.126	10.45 ± 2.7	12.57 ± 3.38	8.02 ± 2.357	90.85 ± 10.6
Test	*t* = −0.335 *p* = 0.738	***t* = −2.338** ***p* = 0.020**	*t* = 0.837 *p* = 0.403	***t* = −2.576** ***p* = 0.010**	*t* = 0.326 *p* = 0.744	*t* = 0.507 *p* = 0.612	*t* = 1.434 *p* = 0.152	*t* = 1.321 *p* = 0.187	***t* = 2.172** ***p* = 0.030**
Duration of daily internet use (hours)									
0–1	12.70 ± 5.055	7.00 ± 3.756	10.38 ± 2.396	4.38 ± 2.253	9.76 ± 1.571	10.05 ± 2.8	13.05 ± 3.54	8.86 ± 2.551	93.43 ± 10.1
2–3	12.60 ± 4.443	7.09 ± 3.466	10.31 ± 2.385	4.18 ± 1.969	9.88 ± 2.005	10.43 ± 2.7	13.05 ± 3.20	8.49 ± 2.285	93.51 ± 10.7
4–5	12.74 ± 4.159	6.92 ± 3.485	10.25 ± 2.352	4.31 ± 2.007	9.73 ± 2.115	10.75 ± 2.6	13.14 ± 3.27	8.22 ± 2.211	93.59 ± 10.8
≥ 6	12.17 ± 4.602	7.44 ± 3.843	9.89 ± 2.561	4.36 ± 1.986	9.55 ± 2.133	10.66 ± 2.8	12.63 ± 3.16	7.94 ± 2.380	91.04 ± 11.4
Test	*f* = 0.525 *p* = 0.665	*f* = 0.664 *p* = 0.574	*f* = 1.036 *p* = 0.376	*f* = 0.343 *p* = 0.794	*f* = 0.834 *p* = 0.475	*f* = 1.117 *p* = 0.341	*f* = 0.795 *p* = 0.497	*f* = 2.603 *p* = 0.051	*f* = 1.963 *p* = 0.118
Frequency of searching for online information									
Every day	12.63 ± 4.373	7.05 ± 3.485	10.28 ± 2.321	4.24 ± 1.919	9.75 ± 2.066	10.67 ± 2.675	12.89 ± 3.307	8.21 ± 2.292	93.15 ± 10.734
Several days a week	12.51 ± 4.415	6.85 ± 3.510	10.21 ± 2.526	4.23 ± 2.044	9.77 ± 1.881	10.45 ± 2.751	13.50 ± 2.945	8.59 ± 2.234	93.95 ± 11.343
Once a week	12.15 ± 4.705	8.65 ± 4.270	9.31 ± 2.635	5.04 ± 2.849	9.42 ± 2.969	9.77 ± 2.747	12.58 ± 3.546	8.23 ± 2.732	87.77 ± 11.466
Once a month	12.00 ± 5.117	9.00 ± 4.843	9.17 ± 3.298	4.75 ± 2.261	10.08 ± 1.676	10.17 ± 3.298	11.17 ± 2.949	8.08 ± 2.466	86.92 ± 7.537
Test	*f* = 0.188 *p* = 0.904	***f* = 3.143** ***p* = 0.025**	*f* = 2.110 *p* = 0.098	*f* = 1.577 *p* = 0.194	*f* = 0.333 *p* = 0.802	*f* = 1.204 *p* = 0.307	***f* = 3.042** ***p* = 0.028**	*f* = 1.280 *p* = 0.280	***f* = 3.738** ***p* = 0.011**
Duration of weekly online information searching (hour)									
0–7	12.31 ± 4.462	6.82 ± 3.304	10.36 ± 2.354	4.16 ± 1.915	9.91 ± 1.923	10.67 ± 2.679	13.07 ± 3.250	8.37 ± 2.347	93.70 ± 10.634
8–14	13.05 ± 4.097	6.84 ± 3.288	10.23 ± 2.274	4.30 ± 1.955	9.66 ± 1.979	10.58 ± 2.492	13.01 ± 3.216	8.23 ± 2.195	93.63 ± 10.575
≥ 15	12.91 ± 4.640	8.98 ± 4.685	9.40 ± 2.801	4.79 ± 2.407	9.14 ± 2.651	10.08 ± 3.225	12.62 ± 3.232	8.13 ± 2.276	88.51 ± 12.179
Test	*f* = 2.109 *p* = 0.122	***f* = 14.431** ***p* = 0.000**	***f* = 5.806** ***p* = 0.003**	***f* = 3.648** ***p* = 0.027**	***f* = 5.481** ***p* = 0.004**	*f* = 1.746 *p* = 0.175	*f* = 0.701 *p* = 0.496	*f* = 0.506 *p* = 0.603	***f* = 8.735** ***p* = 0.000**

Abbreviations: *f*, ANOVA; *t*, *t*‐test. The significant values bold represent (p < 0.05).

The examination of the relationship between nursing students' online information searching strategies and their attitudes towards informatics ethical values indicated that attitudes towards informatics ethical values had a statistically significant negative relationship with the disorientation (*r* = −0.241, *p* = 0.000) sub‐dimension and a low‐level statistically significant relationship with the evaluation (*r* = 0.262, *p* = 0.000), purposeful thinking (*r* = 0.254, *p* = 0.000), trial and error (*r* = 0.280, *p* = 0.000), basic ideas differentiation (*r* = 0.276, *p* = 0.000), control (*r* = 0.220, *p* = 0.000) and problem‐solving (*r* = 0.341, *p* = 0.000) sub‐dimensions and online information searching strategies (*r* = 0.339, *p* = 0.000) (Table 4).

**TABLE 4:** Correlation distribution of nursing students' mean scores on the total online information searching strategy scale and the attitudes scale towards informatics ethical values and sub‐dimensions (*n* = 710).

		Disorientation	Evaluation	Purposeful thinking	Trial and error	Basic ideas differentiation	Control	Problem‐solving	Total
Virtual helpfulness	*r*	0.104**	0.148**	0.098**	0.087*	0.091*	0.119**	0.083*	0.096*
	*p*	0.006	0.000	0.009	0.021	0.015	0.002	0.027	0.010
Violation of privacy	*r*	0.318**	−0.123**	−0.146**	−0.168**	−0.168**	−0.116**	−0.254**	−0.233**
	*p*	0.000	0.001	0.000	0.000	0.000	0.002	0.000	0.000
Respect for copyright	*r*	−0.217**	0.105**	0.151**	0.174**	0.142**	0.071	0.204**	0.191**
	*p*	0.000	0.005	0.000	0.000	0.000	0.060	0.000	0.000
Virtual bullying	*r*	0.308**	−0.156**	−0.161**	−0.202**	−0.196**	−0.145**	−0.268**	−0.259**
	*p*	0.000	0.000	0.000	0.000	0.000	0.000	0.000	0.000
Security	*r*	−0.173**	0.028	0.053	0.108**	0.107**	0.018	0.124**	0.108**
	*p*	0.000	0.453	0.160	0.004	0.004	0.628	0.001	0.004
Virtual media collaboration	*r*	−0.049	0.022	−0.025	−0.015	0.019	0.041	0.069	0.028
	*p*	0.195	0.550	0.512	0.682	0.614	0.277	0.068	0.464
Virtual ethics	*r*	−0.120**	0.241**	0.229**	0.260**	0.209**	0.198**	0.230**	0.272**
	*p*	0.001	0.000	0.000	0.000	0.000	0.000	0.000	0.000
Virtual sharing	*r*	0.026	0.139**	0.157**	0.102**	0.146**	0.096*	0.110**	0.132**
	*p*	0.481	0.000	0.000	0.007	0.000	0.010	0.003	0.000
Total	*r*	−0.241**	0.262**	0.254**	0.280**	0.276**	0.220**	0.341**	0.339**
	*p*	0.000	0.000	0.000	0.000	0.000	0.000	0.000	0.000

*Note:* Pearson correlation test was used. The significant values bold represent (p < 0.05).

*p < 0.05; **p < 0.001.

The online information searching strategies had a statistically significant negative relationship with the violation of privacy (*r* = −0.233, *p* = 0.000) and virtual bullying (*r* = −0.259, *p* = 0.000) sub‐dimensions and a positive, low‐level and statistically significant relationship with virtual helpfulness (*r* = 0.096, *p* = 0.010), respect for copyrights (*r* = 0.191, *p* = 0.000), security (*r* = 0.108, *p* = 0.004), virtual ethics (*r* = 0.272, *p* = 0.000) and virtual sharing (*r* = 0.132, *p* = 0.000) sub‐dimensions and attitudes towards informatics ethical values (*r* = 0.339, *p* = 0.000) (Table 4).

## 6 Discussion

Due to the frequent use of up‐to‐date technology in the field of health, it is important for nursing students to access online information easily and quickly to have professional competence. However, information technologies used to access online information also bring ethical problems. In this section, the findings obtained from our research, which was conducted to examine nursing students' online information searching strategies and attitudes towards informatics ethical values and the relationship between the two, are discussed in light of the literature.

### 6.1 The Comparison of Nursing Students' Online Information Searching Strategies According to Their Demographic Characteristics

In our study, it was determined that female participants' online information searching strategy levels were statistically significantly higher than those of males. In contrast, Ay and Seferoğlu [30] determined that male students' online information searching strategies were more significantly developed than those of females. In the literature, it has been reported that there is no relationship between online information searching strategies and gender in studies consisting of nursing students [2] and different sample groups [31]. It is thought that the similarities and differences between our findings and those in the literature may have been due to the differences in the sample groups and data collection tools used in the studies. These discrepancies may be due to demographic differences in the sample groups, cultural factors and the variety of data collection tools used. In addition, the effect of gender on online information‐seeking strategies is shaped by factors, such as digital skill levels and frequency of internet access. In this context, the development of digital skills may affect the online information‐seeking strategies of women and men, and cultural contexts also play an important role in determining these differences.

Gender differences are even more important in terms of problem‐solving skills in nursing education [32]. In our study, it was determined that female participants had higher problem‐solving levels than male participants. Unlike our study results, there are also results in the literature showing that male students' problem‐solving skills are higher than those of female students [30]. It has also been claimed that there is no relationship between gender and problem‐solving behaviours [32]. It is thought that the differences may have arisen from diversity in geographical regions, cultures, gender roles, nursing education styles and institutional policies.

In our study, online information searching strategies differed according to the status of having a personal computer, and it was determined that the online information searching strategy levels of those who had a computer were higher. No similar study was found in the literature. It is thought that having a personal computer may have increased the level of participants' information searching skills due to frequent access to the Internet. According to our study results, participants with good or very good levels of computer use had higher levels of evaluation, purposeful thinking, control, problem‐solving, trial and error, and online information searching strategies. In another similar study, it was stated that students with advanced levels of computer use had higher levels of control, problem‐solving, disorientation, purposeful thinking, online information searching strategies, and trial and error [30]. In line with this information, considering that more than half of the students participating in our study had moderate, good or very good computer use levels, it can be said that they were more comfortable in searching for online information and could achieve their goals and get important information on the web.

### 6.2 Discussion of Nursing Students' Attitudes Towards Informatics Ethical Values According to Their Demographic Characteristics

Individuals' ethical perceptions and status of exhibiting unethical behaviour were affected by age and school year variables [26]. According to our research results, there was a significant relationship between students' age and school year and their attitudes towards informatics ethics. Yanar et al. [26] stated that participants' scores on the attitude towards informatics ethical values increased with age. Kurt and Teker [27] found that as the school year of students increased, their ethical behaviour scores also increased. Nursing education aims to develop professionalisation and ethical attitudes in students as the age and school year progress [33, 34]. In our study, female participants had lower attitudes towards violation of privacy and virtual bullying than males, but their general attitudes towards respect for copyrights, security, virtual ethics and informatics ethics were found to be higher. Another similar study showed that females exhibited more ethical behaviour towards informatics than males [26]. The study results of Kurt and Teker [27] support our findings. The emotional intelligence of female nursing students is higher than that of males, making them more empathetic [32]. In addition, considering the differences in emotional functioning mechanisms between men and women, it is thought that women's ethical attitudes and judgments may be more developed.

According to our findings, students who had a personal computer had higher general attitude levels towards informatics ethics. In addition, nursing students' general attitude levels towards informatics ethics differed according to the duration and frequency of searching for information on the Internet. Those who searched for information online every day and several times a week for 15 h or more had lower general attitude levels towards virtual ethics and informatics ethics. In another study, it was stated that prospective teachers' ability to use the Internet was an important predictor of their internet ethics behaviour but that the total internet use experience and the average time they spent using the Internet a day did not have a significant effect on predicting their internet ethics behaviour [27].

According to our study results, nursing students' general attitude levels towards informatics ethics differed according to their school year and the high school they graduated from. There was limited research in the literature related to this finding. In a study conducted with high school students, it was stated that students' informatics ethics scores did not differ significantly according to their school year, but a difference was found according to the school they graduated from [35]. All these results may lead to the interpretation that students' needs for informatics ethics differ according to their age, school year and cognitive competence levels. Additionally, students may have been exposed to different informatics ethics issues in institutions providing pre‐university education. In this regard, it is clear that the profiles of the schools from which students graduate should also be taken into account when deciding on informatics ethics issues for those in different school years.

### 6.3 Discussion of the Relationship Between Nursing Students' Online Information Searching Strategies and Attitudes Towards Informatics Ethical Values

There was no study in the literature on the investigation and discussion of attitudes towards informatics ethical values and online information searching strategies together. Studies in the international literature mostly focused on health informatics, and the future of health informatics, especially the post‐COVID‐19 period, was emphasised [36]. They also included the examination of informatics applications, such as artificial intelligence in terms of ethical values [37]. Additionally, ethical issues faced by nurses [38] and patients [39] in the field of healthcare were emphasised. A very limited number of studies on the examination of ethical issues in nursing informatics were found [40]. No studies on the examination of nursing students' informatics ethical values and online information searching strategies together were encountered. According to our study results, a significant relationship was found between nursing students' general attitudes towards informatics ethics and online information searching strategies. As students' online information searching strategies increased, their attitudes towards informatics ethical values also increased. Digital technology tools increase the quality of nursing education and enable students to adapt to modern healthcare practices. These tools help students gain practical skills and prepare for real‐world healthcare environments [41]. Because it will increase the speed of access to information, its use in decision‐making processes in clinical areas will be beneficial in interpreting patient results and accessing high‐level evidence for appropriate care interventions [42, 43]. Integrating technology into teaching processes is important for training successful nursing students in digital healthcare [44]. Providing academic and logical support for the digital transformation of nursing programmes can provide valuable knowledge and practical insights for the future of nursing education [45]. To sustain quality and evidence‐based care and education in nursing, informatics and technology must be included in the undergraduate nursing curriculum. In this way, students' digital literacy and information searching skills will improve in both university and clinical environments [5, 40]. In addition, addressing the issues encountered with digital literacy and integrating appropriate content into the curriculum will enable better quality patient care and effective use of data in various healthcare settings [41].

### 6.4 Study Limitations

One of our limitations is that our study was conducted at a single university because of the researchers' conditions. In addition, the results may have been affected by cultural and regional differences. The study data were based on students' self‐reporting and collected using a non‐probability sampling method. Another limitation is that the cross‐sectional design type does not allow causal conclusions. For these reasons, it is not possible to generalise the results obtained from the study. The results suggest that future studies could expand their sample to multiple institutions or countries to increase generalisability.

## 7 Conclusion

According to our study results, nursing students' age, gender, the status of having a personal computer, level of computer use, status of having an internet connection, the duration of weekly online information searching and frequency of information searching on the Internet affected their online information searching strategies and general attitudes towards informatics ethics. In addition, as students' attitudes towards informatics ethical values increased, their online information searching strategies also increased. Based on the study findings, some recommendations for education, research and clinical practice are made.

In the field of education, it is recommended that higher education institutions make the necessary plans to develop online information search strategies and raise awareness about informatics ethics throughout the educational life of students. It is particularly recommended that information ethics be included in the curriculum. The strategies that students use in digital environments should be revealed throughout the educational period, and courses can be planned to develop them. Universities need to inform students about laws related to informatics ethics. In this context, it is important for higher education institutions to strengthen their information systems that can be used in theoretical, laboratory and clinical teaching. In‐service training and seminars should be provided to students who start working in the health field after graduation on online information search strategies and informatics ethics.

Nowadays, the importance of developing infrastructures related to online information search and the use of online tools in research has increased. It has become clear that future studies are needed, and it is thought that this process can be examined in more detail with qualitative studies. In addition, the study can be repeated in different countries and institutions with large samples.

Based on our study results, it is thought that the subject is also important in the field of clinical practice. It is assumed that increasing digital literacy and ethical informatics education will increase the quality of patient care, privacy and data security in clinical settings. In this context, studies are recommended to examine the effects of online information search and informatics ethics education given to students and the effects on patient care outcomes after education.

## Author Contributions

**Necibe Dagcan Sahin:** conceptualisation, methodology, software, reviewing, editing. **Gulsah Gurol Arslan:** conceptualisation, supervision, data curation, writing – original draft preparation. **Dilek Ozden:** conceptualisation, data curation, writing – original draft preparation. There is a statistician on the author team and state which author: Necibe Dagcan Sahin. The author(s) affirm that the methods used in the data analyses are suitably applied to their data within their study design and context, and the statistical findings have been implemented and interpreted correctly. The author(s) agrees to take responsibility for ensuring that the choice of statistical approach is appropriate and is conducted and interpreted correctly as a condition to submit to the journal.

## Ethics Statement

The authors developing the scales used for data collection were asked for their permission. Informed consent was obtained from the students and permission was taken from the faculty of nursing where the study was carried out. Also, ethical approval was obtained from the ethical board of the university (Dokuz Eylül University Non‐Interventional Ethics Committee, date: June 23, 2021; decision number: 2021/19‐11).

## Conflicts of Interest

The authors declare no conflicts of interest.

## Data Availability

The data that support the findings of this study are available from the corresponding author upon reasonable request.

## References

[1] A. Gecer and N. Ira , “Examining Information in Web Environment Searching and Commitment Strategies of University Students According to Demographic Variables,” Education in Science 40, no. 179 (2015): 383–402, 10.15390/EB.2015.3131.

[2] G. Ozden , S. Cevik , and S. Citlik Saritas , “Do the Online Information Searching Strategies Affect Individual Innovativeness in Nursing Students?,” Annals of Medical Research 26, no. 4 (2021): 629–635, 10.5455/annalsmedres.2019.01.018.

[3] G. Goktuna , G. Gurol Arslan , and D. Ozden , “Researches on Nursing Informatics in Turkey: A Literature Review,” Medical Science 15, no. 4 (2020): 99–110, 10.12739/NWSA.2020.15.4.1B0094.

[4] Z. Tatlı , A. Aydın , P. Şimşek , et al., “The Use of Information Technologies by Nurses and Nursing Students,” Ordu University Journal of Nursing Studies 1, no. 1 (2018): 18–27.

[5] A. Harerimana , K. Wicking , N. Biedermann , and K. Yates , “Nursing Informatics in Undergraduate Nursing Education in Australia Before COVID‐19: A Scoping Review,” Collegian 29, no. 4 (2022): 527–539, 10.1016/j.colegn.2021.11.004. 34867065 PMC8626237

[6] G. Türk , “Chapter 6: Nursing Theories and Models,” in Essentials of Nursing Practice (Temel Hemşirelik Uygulama İçin Esaslar), ed. Ş. Karagözoğlu , A. Demiray , and P. Doğan (Nobel Tıp Kitabevleri, 2023), 83–85.

[7] E. Sarıtaş and Z. Göçmen Baykara , “Chapter 3: Care and Nursing,” in Essentials of Nursing Practice (Temel Hemşirelik Uygulama İçin Esaslar), ed. Ş. Karagözoğlu , A. Demiray , and P. Doğan (Nobel Tıp Kitabevleri, 2023), 244–249.

[8] R. M. Neil and M. A. Tomey , “Jean Watson: Philosophy and Science of Caring,” in Nursing Theorists and Their Work, 6th ed., ed. A. M. Tomey and M. R. Alligood (Mosby Inc, 2006).

[9] L. Fredriksson and K. Eriksson , “The Ethics of the Caring Conversation,” Nursing Ethics 10, no. 2 (2003): 138–148, 10.1191/0969733003ne588oa. 12659485

[10] G. Senay , “Nursing Care in the Light of Care Concept and Affecting Factors (Bakım Kavramı Işığında Hemşirelik Bakımı ve Etkileyen Faktörler),” Acıbadem University Health Sciences Journal 10, no. 2 (2019): 129–134.

[11] C. Ayik and G. G. Arslan , “Effectiveness of Caring Behaviours Course on Decision‐Making and Caring Behaviours in Undergraduate Nursing Students: An Experimental Study,” Scandinavian Journal of Caring Sciences 38, no. 4 (2024): 864–875, 10.1111/scs.13288. 39092534

[12] D. Bayraktar , A. K. Aydın , T. Eliş , and K. Öztürk , “Inclination of Nursing Students Towards Ethical Values and the Effects of Ethical Values on Their Care Behaviours,” Journal of Bioethical Inquiry 20, no. 3 (2023): 433–445, 10.1007/s11673-023-10269-0. 37402121

[13] F. Shirazi , S. Heidari , S. J. Fard , F. Ghodsbin , and M. R. Koohpeyma , “Pattern of Internet Use by Iranian Nursing Students: Facilitators and Barriers,” Investigation Education Enfermeria 37, no. 2 (2019): e06, 10.17533/udea.iee.v37n2e06. PMC7871486 31487443

[14] E. Senyuva , “Relationship Between the Internet Self‐Sufficiency Levels and Online Information Search Strategies of Nursing Students,” Journal of Turkish Science Education 15, no. 2 (2017): 102–116.

[15] C. Çaka , E. Doğan Barut , and Y. Şahin , “The Examination of Online Information Searching Strategies of Students That Use Social Network (Sosyal Ağ Kullanan Öğrencilerin Çevrimiçi Bilgi Arama Stratejilerinin Çeşitli Değişkenler Açısından İncelenmesi),” Trakya Journal of Education 6, no. 1 (2015): 1–13.

[16] M. Özlü and F. Kantek , “Interventions to Increase Nurses' Motivation: Systematic Review (Hemşirelerin Motivasyonunu Artırmaya Yönelik Müdahaleler: Sistematik Inceleme),” Journal of Research and Development in Nursing 26, no. 3 (2024): 23–38, 10.69487/hemarge.1491057.

[17] Y. D. Arikan and S. H. Duymaz , “Practice of Information Technology Ethics Education,” Elementary Education Online 14, no. 1 (2015): 188–199, 10.17051/io.2015.92430.

[18] B. Celik and K. Gundogdu , “Developing an Attitude Scale Towards Informatics Ethical Values,” Ege Journal of Education 20, no. 2 (2019): 335–350, 10.12984/egeefd.590560.

[19] A. A. Kurt and B. G. Emiroglu , “Analysis of Students' Online Information Searching Strategies, Exposure to Internet Information Pollution and Cognitive Absorption Levels Based on Various Variables,” Malaysian Online Journal of Educational Technology 6, no. 1 (2017): 18–29.

[20] P. Askar and S. G. Mazman Akar , “Adaptation of Online Information Searching Strategy Inventory Into Turkish,” Education in Science 38, no. 168 (2013): 167–182.

[21] G. Rouleau , M. P. Gagnon , J. Côté , J. Payne‐Gagnon , E. Hudson , and C. A. Dubois , “Impact of Information and Communication Technologies on Nursing Care: Results of an Overview of Systematic Reviews,” Journal of Medical Internet Research 19, no. 4 (2017): e122, 10.2196/jmir.6686. 28442454 PMC5424122

[22] Z. Bozok , E. Geniş , and Y. Avcu , “Development and Implementation of A Digital Game for Information Technology (IT) Ethics Teaching in Gifted and Talented Students,” International Journal of Education Science and Technology 6, no. 1 (2020): 36–54.

[23] B. Gökçay and B. Arda , “Ethical Overview of Health Research With Regard to the Protection of Personal Health Data (Kişisel Sağlık Verilerinin Korunması Kapsamında Sağlık Araştırmalarında Etik Bakış),” Archives of the Turkish Society of Cardiology 47, no. 3 (2019): 218–227. 30982810 10.5543/tkda.2019.15957

[24] E. Mutluay and L. Özdemir , “Use of Nursing Informatics Within the Scope of Health Information Systems (Sağlık Bilişim Sistemleri Kapsamında Hemşirelik Bilişiminin Kullanımı),” Florence Nightingale Journal of Nursing 22, no. 3 (2014): 180–186.

[25] E. Abdoh , “Online Health Information Seeking and Digital Health Literacy Among Information and Learning Resources Undergraduate Students,” Journal of Academic of Librarianship 48, no. 6 (2022): 102603, 10.1016/j.acalib.2022.102603. PMC9487174 36158639

[26] N. Yanar , G. Ceylan , and S. Tuncel , “Examination of Ethical Behavior in Distance Education Process of Undergraduate Students Who Take and Do Not Take Ethics Course,” International Journal of Contemporary Educational Studies 7, no. 1 (2021): 111–125.

[27] M. Kurt and N. Teker , “Identifying Preservice Teachers' Netiquette Behaviors,” Bartın University Journal of Faculty of Education 6, no. 2 (2017): 749–769, 10.14686/buefad.315239.

[28] S. Kılıç , “Sampling Methods (Örnekleme Yöntemleri),” Journal of Mood Disorders 3, no. 1 (2013): 44–46, 10.5455/jmood.20130325011730.

[29] M. J. Tsai , “Online Information Searching Strategy Inventory (OISSI): A Quick Version and a Complete Version,” Computers in Education 53, no. 2 (2009): 473–483.

[30] K. Ay and S. S. Seferoğlu , “The Investigation of Online Information Search Strategies of Graduate Students in Terms of Various Variables,” Kastamonu Education Journal 25, no. 1 (2017): 51–66, 10.24106/Kefdergi.308051.

[31] Z. Turan , İ. Reisoğlu , E. Özçelik , and Y. Göktaş , “The Examination of Teacher's Online Information Searching Strategies in Terms of Different Variables,” Kastamonu Education Journal 23, no. 2 (2015): 1–16.

[32] X. Deng , S. Chen , X. Li , et al., “Gender Differences in Empathy, Emotional Intelligence and Problem‐Solving Ability Among Nursing Students: A Cross‐Sectional Study,” Nurse Education Today 120 (2023): 105649, 10.1016/j.nedt.2022.105649. 36435156

[33] B. Ak , “Professional Attitudes of the Nursing Students and Associated Factors,” Journal of Contemporary Medical Education 27, no. 4 (2018): 232–234.

[34] E. Bayrak Aykan , B. Eren Fidancı , and D. Yıldız , “Evaluation of Moral Maturity and Ethical Sensitivity in Nursing Students,” University of Health Sciences Journal of Nursing 1, no. 2 (2019): 84–91.

[35] E. Bayra and E. Baysan , “Analysation of Information Ethics Levels of Secondary School Students in Terms of Some Variables With Real‐Life Scenarios,” Gazi Journal of Educational Science 8, no. 1 (2022): 82–107, 10.30855/gjes.2022.08.01.006.

[36] M. Sallam , N. A. Salim , A. B. Al‐Tammemi , et al., “ChatGPT Output Regarding Compulsory Vaccination and COVID‐19 Vaccine Conspiracy: A Descriptive Study at the Outset of a Paradigm Shift in Online Search for Information,” Cureus 15, no. 2 (2023): e35029, 10.7759/cureus.35029. 36819954 PMC9931398

[37] J. C. De Gagne , H. Hwang , and D. Jung , “Cyberethics in Nursing Education: Ethical Implications of Artificial Intelligence,” Nursing Ethics 31, no. 6 (2024): 9697330231201901, 10.1177/09697330231201901. 37803810

[38] P. Snelling , “Am I My Students' Nurse? Reflections on the Nursing Ethics of Nursing Education,” Nursing Ethics 31, no. 1 (2024): 52–64, 10.1177/09697330231193858. 37769641 PMC10898194

[39] H. Zendehtalab , Z. Vanaki , and R. Memarian , “Ethical Challenges in Caring for Healthy Older Adults: Qualitative Perspectives,” Nursing Ethics 30, no. 4 (2023): 542–555, 10.1177/09697330221081953. 36841931

[40] J. Wang , Y. Xu , X. Zhang , and H. Pan , “Ethical Predicaments and Countermeasures in Nursing Informatics,” Nursing Ethics 31 (2023): 1050–1064, 10.1177/09697330231215962. 37976551

[41] J. Brown , A. Morgan , J. Mason , N. Pope , and A. M. Bosco , “Student Nurses' Digital Literacy Levels: Lessons for Curricula,” Computers, Informatics, Nursing 38, no. 9 (2020): 451–458, 10.1097/CIN.0000000000000615. 33955370

[42] B. Kahveci Ceylan and M. Mete , “The Concept of Evidence‐Based Nursing and Evidencegeneration Situations (Kanıta Dayalı Hemşirelik Kavramı ve Kanıt Niteliği Oluşturan Durumlar),” Selçuk Üniversitesi Akşehir Meslek Yüksekokulu Sosyal Bilimler Dergisi 2023, no. 15 (2023): 121–132.

[43] K. K. Fung , S. S. Hung , D. W. L. Lai , M. H. Y. Shum , H. W. Fung , and L. He , “Access to Information and Communication Technology, Digital Skills, and Perceived Well‐Being Among Older Adults in Hong Kong,” International Journal of Environmental Research and Public Health 20, no. 13 (2023): 6208, 10.3390/ijerph20136208. 37444058 PMC10340767

[44] A. A. G. Nes , S. A. Steindal , M. H. Larsen , H. C. Heer , E. Lærum‐Onsager , and E. R. Gjevjon , “Technological Literacy in Nursing Education: A Scoping Review,” Journal of Professional Nursing 37, no. 2 (2021): 320–334, 10.1016/j.profnurs.2021.01.008. 33867086

[45] P. Li , R. Tan , T. Yang , and L. Meng , “Current Status and Associated Factors of Digital Literacy Among Academic Nurse Educators: A Cross‐Sectional Study,” BMC Medical Education 25, no. 1 (2025): 16, 10.1186/s12909-024-06624-3. 39754150 PMC11697852

